# Deep Active Learning for Surface Defect Detection

**DOI:** 10.3390/s20061650

**Published:** 2020-03-16

**Authors:** Xiaoming Lv, Fajie Duan, Jia-Jia Jiang, Xiao Fu, Lin Gan

**Affiliations:** The State Key Lab of Precision Measuring Technology and Instruments, Tianjin University, Tianjin 300072, China; lvxiaoming1@gmail.com (X.L.); jiajiajiang@tju.edu.cn (J.-J.J.); fuxiao215@tju.edu.cn (X.F.); ganlin@tju.edu.cn (L.G.)

**Keywords:** surface defect detection, active learning, deep learning

## Abstract

Most of the current object detection approaches deliver competitive results with an assumption that a large number of labeled data are generally available and can be fed into a deep network at once. However, due to expensive labeling efforts, it is difficult to deploy the object detection systems into more complex and challenging real-world environments, especially for defect detection in real industries. In order to reduce the labeling efforts, this study proposes an active learning framework for defect detection. First, an Uncertainty Sampling is proposed to produce the candidate list for annotation. Uncertain images can provide more informative knowledge for the learning process. Then, an Average Margin method is designed to set the sampling scale for each defect category. In addition, an iterative pattern of training and selection is adopted to train an effective detection model. Extensive experiments demonstrate that the proposed method can render the required performance with fewer labeled data.

## 1. Introduction

Defects may appear on metallic surfaces due to irresistible influencing factors, such as material characteristics and processing technologies in industrial production. These defects affect not only the qualities, but also the applications of products. Thus, it is of great significance to detect such defects for quality control. As the main solution for defect detection, image processing techniques for detection generally consist of defect localization, recognition, and classification. These methods can be roughly divided into two categories: Traditional image processing and deep learning.

In traditional industries, traditional image processing is usually adopted as a defect detection method combined with hand-crafted features [1], which are exploited to describe the defects. In addition, these approaches require many complex threshold settings, which are sensitive to changes in real-world environments and easily influenced by illumination, background, and so on. Therefore, these approaches are easily affected by environmental noise, which may lead to a poor detection model with weak performance. At the same time, traditional algorithms are weak in processing speeds and cannot meet the requirements of real-time detection. Furthermore, they lack robustness for real-world deployments due to their weak adaption capabilities.

With the rapid development of deep learning, convolutional neural networks (CNN) have been successfully implemented for metallic surface defect detection [2]. For example, Ri-Xian et al. [3] designed a deep confidence network (DCN) for defect detection. Masci et al. [4] proposed a defect detection model for steel classification to reduce the processing time via CNN with a maximum pool. Liu et al. [5] added a feature extraction module based on Faster-RCNN for defect detection. In addition, regional suggestion and regression operation are also widely used in deep learning for defect detection. The former is to generate candidate defect boxes that are classified by CNNs. For example, Girshick developed a defect detection method based on Fast-RCNN [6] to detect five types of defects. The latter can directly perform on the target bounding boxes, such as OverFeat [7,8], SSD [9], YOLO-v2 [10], and YOLO-v3 [11]. OverFeat is a network for integrating recognition, localization, and detection using convolutional networks. The YOLO-v1 method is the base network for YOLO-v3 and YOLO-v3. YOLO-v2 added batch normalization, a high-resolution classifier, multi-scale training, and dimension clusters, while YOLO-v3 used an optimized darknet network and multi-scale prediction. In addition, SSD adopted a softmax classifier, while YOLO methods adopted a logistic classifier. However, all of these approaches assume that a large number of labeled images are available, which require expensive and time-consuming labeling efforts. This problem significantly hinders the deployment of the deep detection models into real-world industries.

As an important branch of machine learning for reducing labeling efforts, active learning has been used to solve data collection problems for many applications. There are situations where unlabeled images are abundant, but manually labeling is expensive. In such a scenario, active learning algorithms can actively query the annotators for labels. Active learning algorithms are mainly aimed at selecting effective data for annotation. Thus, the number of images can often be much lower than the number required in supervised learning. The common query strategies include: (1) Expected model change: Annotate images that would change the current model the most. (2) Uncertainty sampling: Annotate images for which the current model is least certain. (3) Variance reduction: Annotate images that would minimize output variance, which is one of the components of error. Theoretical results indicate that a great active selection strategy can significantly reduce the labeling efforts compared to random selection for obtaining similar accuracy. Although the existing active selection strategies have demonstrated great performance in deep learning, these strategies cannot be directly employed in defect defection. Thus, the active learning for defect detection still faces large challenges. Compared with existing deep detection methods [12,13], active learning methods try to train an effective detection model with the least labeling effort, while deep learning methods annotate all data and train the model with the full training set. Uncertainty is the main problem of performance measurement for deep models, so the active learning methods can select the valuable data for querying annotations.

To effectively reduce the labeling efforts for defect detection, this research introduces active learning into metallic defect detection via uncertainty of data. First, based on the information of the defect images, we propose an Uncertainty Sampling to the product candidate list for annotation. Then, to estimate the sampling scale, an Average Margin method is designed for scale calculation. Finally, a series of experiments is conducted to demonstrate the effectiveness of this method on the NEU-DET [14] dataset.

## 2. Related Work

### 2.1. Object Detection for Defect Detection

Over recent years, deep metallic surface defect detection methods have obtained relatively outstanding results in single-image backgrounds [15]. At the same time, deep object detection approaches are the main methods for detecting defects, such as YOLO [8,10,11], Faster-RCNN [6], and SSD [9].

To meet the real-time requirement of object detection, Radmond et al. [8] proposed the YOLO-v1 algorithm to reduce computational complexity and found that end-to-end real-time monitoring is feasible. Meanwhile, combined with machine learning, the extraction of physical information and the construction of structural features show more advantages in the field of defect detection, making a shallow-to-deep transition. Therefore, deep learning plays a crucial role in defect detection at present. Many defect detection methods are proposed based on deep object detection. For example, Feng et al. [16] built an improved version based on the YOLO model, which is widely used in production monitoring, mobile location, and surface defect detection. Ri-Xian et al. [3] proposed to use a convolutional neural network (CNN) to detect defects on the surfaces by trying to overcome the overfitting problem of small data sets that often occur in practical applications. Ren et al. [17] proposed a surface defect detection model for automatic detection. Azimi et al. [18] used a fully convolutional network for pixel-level segmentation. However, these methods require huge numbers of labeled images, which shall be collected in real-world industries and annotated by great human labors. Thus, it is difficult to deploy them in real-world industries due to the expensive labeling efforts.

### 2.2. Active Learning

Active learning is a popular approach for reducing the labeling efforts. Its core problem is the sampling strategy, which is used to estimate the labeling value of unlabeled data. Differently from the traditional classifiers that are trained on a large number of data, active learning can speed up the convergence of detection models and reduce the number of labeled data. To effectively reduce the labeling efforts, active learning often selects the samples containing abundant information via specially designed query strategies.

A common active strategy form is the committee [19], which is used to construct a similar classifier for data selection. Then, the voting results are produced by multiple classifiers. In this setting, the samples with the greatest divergence or complexity are selected and labeled. For example, Tuia et al. [20] suggested a simple way to select samples based on the uncertainty: First, all samples are tested by a classifier, and then the most uncertain samples are given to experts for annotations. Xu et al. [21] proposed a density-weighting method to select samples with the richest information based on the potential characteristics of the data. Chakraborty et al. [22] proposed that numerical-optimization-based techniques can also help the selection of useful samples. In addition, Tong et al. [19] proposed a kernel-space-based clustering algorithm for sample selection by using an RBF (radial basis kernel function) to map samples into several clusters in a high-dimensional space, and then selecting the center samples. However, these methods merely consider data characteristics, ignoring the training process of the deep models.

Although defect detection methods based on deep learning have been well studied and greatly improved, there are still expensive and time-consuming labeling efforts for dataset building. Thus, it is important to take the reduction of labeling efforts into consideration for defect detection.

## 3. Active Learning for Defect Detection

The following sections first introduce an overview of the proposed active learning framework, and then describe the detection model used in the framework in detail. Furthermore, we illustrate the two main components of the framework, i.e., Uncertainty Sampling for Candidates and the Average Margin for Scale.

### 3.1. Overall Framework

**Figure 1:**
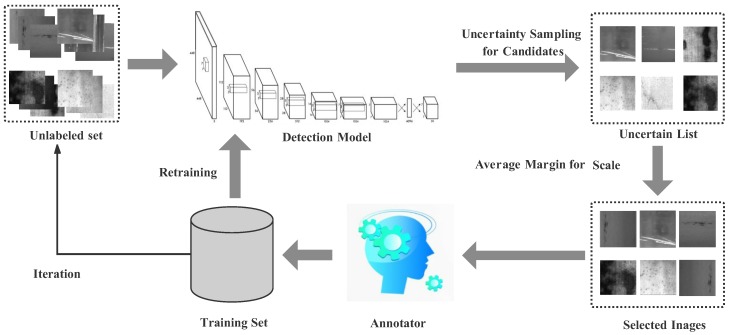
Overview of the proposed active learning framework. First, a defect detection model (neural network) is initialized via pre-training weights. Second, unlabeled images are fed into the detection model for prediction results. Then, combined with the proposed strategies, uncertain images are selected for querying annotations. After being labeled by annotators, the images can be added into the training set, and the detection model is updated from scratch. The above steps are repeated until the required performance is achieved.

To reduce the labeling efforts in defect detection, we propose an active learning framework that consists of three main modules: The detection model, active strategies, and data. As shown in Figure 1, it utilizes an iterative pattern for selection and annotation. First, a defect detection model is initialized via pre-training weights. Second, the unlabeled images are fed into the detection model for prediction results. Then, combined with the proposed strategies, the uncertain images are selected for querying annotations. After being labeled by annotators, these images are added into the training set, and the detection model is updated from scratch. The above steps are repeated until the required performance is achieved.

### 3.2. Detection Model

**Figure 2:**
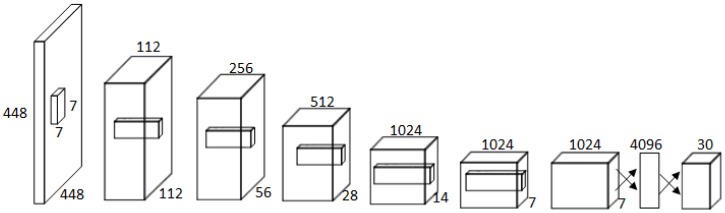
Architecture of the YOLO-v2 model. This network has 24 convolutional layers followed by 2 fully connected layers. Alternating 1×1 convolutional layers reduce the feature space from preceding layers. We pretrain the convolutional layers on the ImageNet classification task at half of the resolution (224×224 input image) and then double the resolution for detection.

Differently from other object detection methods, YOLO-v2 [23] integrates region prediction and object classification into a single neural network. As illustrated in Figure 2, YOLO-v2 includes a convolutional layer of 3×3 kernel size and a sampling window with a size of 2×2. In addition, the object detection task can be regarded as a regression problem for object region prediction and classification. YOLO-v2 utilizes a single network to directly predict the object boundary and classification probability in an end-to-end way. It adds a batch normalization for each convolution layer and adopts a high-resolution classifier (448×448). In addition, YOLO-v2 introduces anchor boxes to predict bounding boxes.

### 3.3. Active Learning for Detection

#### 3.3.1. Uncertainty Sampling for Candidates

In this section, we propose an Uncertainty Sampling for Candidates (USC) algorithm to produce the candidate list for annotation. To estimate the uncertainty of each unlabeled sample, the prediction probabilities for classification are used to calculate the value. Aimed at selecting the most valuable samples for annotation, the outputted probabilities are denoted as follows:

(1) $$P\left(x_{i}\right)=\left\{p\left(y_{i}=1|x_{i};W\right),p\left(y_{i}=2|x_{i};W\right),\ldots ,p\left(y_{i}=n|x_{i};W\right)\right\},$$

where *n* is the number of the defect categories.

Note that if the performance of the model is great enough, the model can accurately detect and classify defect images. In this case, the distribution of predicted probabilities has the following characteristics:

- A higher predicted value denotes the higher probability belonging to the corresponding defect.
- There is the maximum value that denotes the probability belonging to the corresponding defect.
- The probability belonging to the corresponding defect is higher than other probabilities of the remaining defects.

To select the most valuable samples, we need to select the sample with inaccurate predictions. According to the above items, we design a predicted margin strategy to estimate the uncertainty of each unlabeled sample, which can be written as:

(2) $$Margin\left(x_{i}\right)=p\left(y_{i}=j_{max}|x_{i};W\right)-p\left(y_{i}=j_{smax}|x_{i};W\right),$$

where $p\left(y_{i}=j_{max}|x_{i};W\right)$ is the maximum probability, $p\left(y_{i}=j_{smax}|x_{i};W\right)$ is the second maximum probability $x_{i}$, and *W* is the weights of the model.

The predicted margin strategy means that, if a sample is more uncertain, the distribution of predicted probabilities is more average. Thus, the margins among predicted probabilities are small for an uncertain sample. In this case, we select maximum probability and the second one to measure the uncertainty of an unlabeled sample. The smaller value denotes the larger uncertainty of the sample.

#### 3.3.2. Average Margin for Scale

In this section, we design an Average Margin for Scale (AMS) method to measure the sampling scale of the different defects. In fact, a more uncertain defect category needs more unlabeled samples for annotation. Thus, to measure the uncertainty of each defect category, we calculate the average predicted margin for each category. Denote $p(y_{i}=j|x_{i};W)$ as the probability of $x_{i}$ belonging to the *j*-th defect category. The average margin for each category is calculated as follows:

(3) $$Magin_{c}^{avg}=\frac{1}{N}*\sum_{i=1}^{N}Margin_{x_{i}},$$

where *N* is the total sample number of the *c*-th category in the testing set.

The final sample scale can be decided by:

(4) $$Scale=Magin_{1}^{avg}:Magin_{2}^{avg}:\ldots :Magin_{C}^{avg},$$

where $Magin_{c}^{avg}$ is the mean uncertainty of the *c*-th category in the testing set, and *C* is the number of defect categories.

In addition, when selecting uncertain samples as in the above sampling scale, the samples are ranked in the ascending order.

#### 3.3.3. Overview Sampling Algorithm

First, the detection model is initialized by some randomly labeled samples. Then, we calculate the $Margin_{x}$ value for each unlabeled sample. In addition, the average margin for each category $Magin^{avg}$ in the testing set is calculated. Then, unlabeled samples of each category are ranked in the ascending order. Finally, we select samples according to the scale of $Magin^{avg}$. The algorithm can be referred to Algorithm 1.
**Algorithm 1:** Active Learning for Defect Detection.
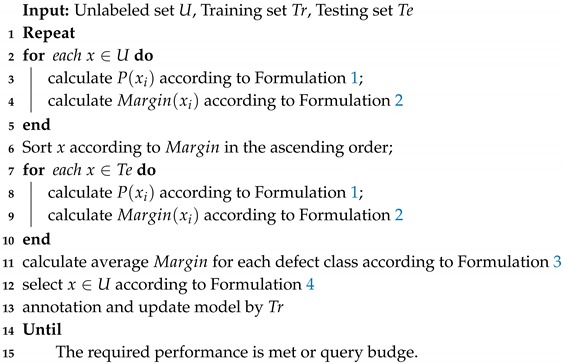


## 4. Experiments

To train an effective detection model, we adopted a small-batch gradient descent method to update the learning rate. The momentum can speed up the convergence of the model, and it is set as 0.9. At the same time, the pre-trained weights are used for the training, and the initialized learning rate was set at 0.005.

### 4.1. Dataset Introduction

**Figure 3:**
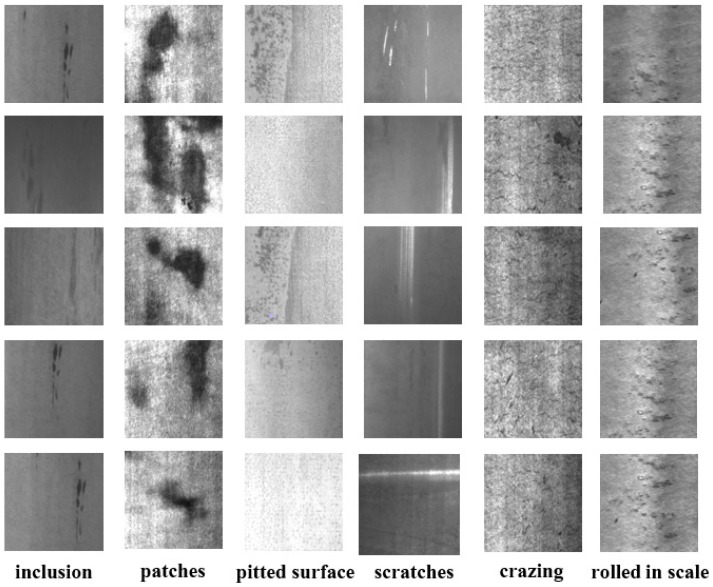
Typical surface defect samples in the NEU-DET dataset.

A surface defect dataset called NEU-DET [14] was published by Northeastern University, which collected six typical surface defects on metal surfaces, as shown in Figure 3:

The NEU-DET dataset includes six types of surface defects, i.e., crazing (Cr), rolled-in scale (Rs), patches (Pa), pitted surface (Ps), inclusion (In), and scratches (Sc). The dataset contains 1800 grayscale images, and each category contains 300 samples. The dataset also provides complete annotations of defect positions and types.

Due to image quality, we merely utilized a subset of NEU-DET, which includes: Patches (Pa), pitted surface (Ps), inclusion (In), and scratches (Sc). This subset has 1200 samples.

### 4.2. Comparisons

**Random selection**: The random selection (RS) strategy is usually used as a benchmark for the comparison of active learning. In this method, the unlabeled samples are randomly selected in each iteration.

**Entropy selection**: In this method, entropy value ($EN_{i}$) is used to measure the uncertainty of samples. A higher $EN_{i}$ value represents a greater uncertainty for sample *i*, which can be defined as follows:

(5) $$EN_{i}=-\sum_{j=1}^{m}p\left(y_{i}=j|x_{i};W\right)logp\left(y_{i}=j|x_{i};W\right),$$

where *m* is the class number and *p* is the prediction probability for class *j*.

**MS**: In this method, all of the unlabeled samples are ranked in an ascending order according to the $MS_{i}$ value, which can be defined as follows:

(6) $$MS_{i}=p\left(y_{i}=j_{1}|x_{i};W\right)-p\left(y_{i}=j_{2}|x_{2};W\right),$$

where $j_{1}$ and $j_{2}$ represent the first and second most probable class labels predicted by the model, respectively. The MS value denotes the margin. The smaller value means a higher uncertainty of the sample.

### 4.3. Evaluation

We adopt Recall, Average Precision (AP), and mean Average Precision (mAP) for performance evaluation. Recall represents the ratio of correctly detected images and all testing images for each defect category. AP represents the average detected precision for each defect category. mAP is the mean of the average detected precision for all defect categories. In addition, we use the full dataset to train the model to obtain the basic required performance, denoted by "Full". This means that all unlabeled images are annotated to train the model.

### 4.4. Comparison Results

**Figure 4:**
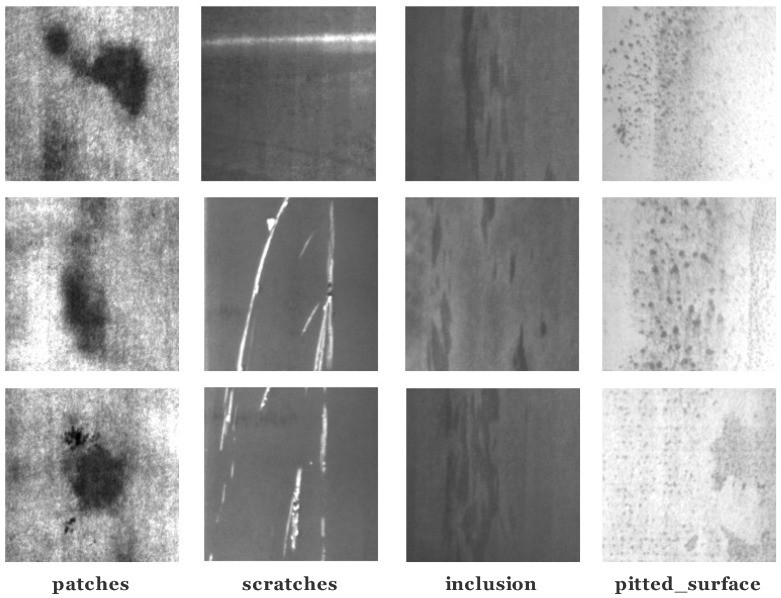
Some images selected by our active learning strategies.

To demonstrate the effectiveness of the proposed method, a series of comparison experiments with Full, MS, EN, and random selection were conducted. From the above experiments, we observed that the performance of the proposed method is better than that of other methods, which indicates the superiority of USC and AMS. Figure 4 exhibits some defect images selected by the active learning strategies of the proposed method.

As illustrated in Table 1, the proposed algorithm can achieve the required Recall accuracy with only 21.7% of the annotations of all datasets. Compared with commonly used active strategies, the proposed algorithm can produce competitive results, i.e., 33.3% for MS, 31.6% for EN, and 50.0% for random. It is noticed that, for defect patches (Pa), pitted surfaces (Ps), and scratches (Sc), the proposed algorithm can outperform the Recall results obtained by the full data. In addition, the MS method performed too badly for the pitted surface (Ps), while performing the best for scratches (Sc).

As shown in Table 2, the proposed algorithm can achieve the required Precision accuracy with only 21.7% of the annotations. In detail, for defect inclusion (In), patches (Pa), pitted surfaces (Ps), and scratches (Sc), our algorithm can outperform the Precision results obtained by both the full data and other active learning methods.

As illustrated in Table 3, the proposed algorithm can achieve the required AP accuracy with only 21.7% of the annotations. In detail, for defect patches (Pa) and pitted surfaces (Ps), the proposed algorithm can get the best AP and mAP results. Although other methods may exceed ours in AP accuracy for several defects, they performed with lower mAP results.

The above experimental results indicate that the proposed method obviously performs better than the commonly used active strategies in terms of both Recall Accuracy and Precision. This is because the proposed method can not only select uncertain samples, but also provide a selection scale for each defect category to train the model. Hence, the proposed method demonstrates a competitive advantage in deep defect detection tasks. To clearly exhibit its effectiveness, we also conducted the performance improvement comparison experiments, and the results are discussed below.

#### 4.4.1. Performance Improvement Comparison

**Figure 5:**
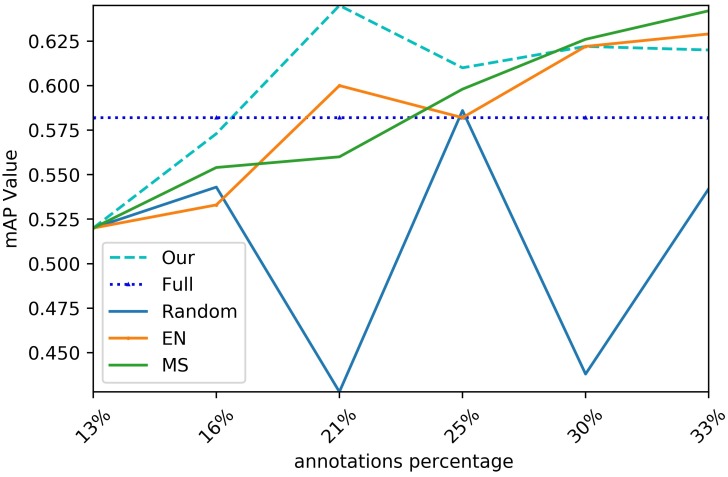
Comparison of mean Average Precision (mAP) for the different strategies.

Figure 5 clearly illustrates the results of the performance improvement comparisons, while Table 4 shows the performance obtained by training on the full data. The horizontal axis represents the annotation percentage, while the vertical axis represents the mAP values. It can be observed that the proposed method can reach the maximum mAP when the percentage is 21.6%, while MS is 33.3% and EN is 31.6%. Therefore, the proposed method performs better than others. This demonstrates that uncertainty sample selection plays an important role in improving the performance.

#### 4.4.2. Query Strategy Analysis

In fact, uncertainty measures the confidence of the current model for a sample, aiming to find the samples with more useful information for model training. The essence of uncertainty is information entropy, which is used to measure the amount of information. A greater information entropy denotes richer information. Thus, many active learning methods [24,25] based on uncertainty are designed to improve the performance of the model and exhibit competitive results.

## 5. Conclusions

In this paper, we propose an active learning framework for a deep defect detection task, which utilizes the uncertainty of the samples for selection. The main contributions of this work are the following three points: (1) We propose an active learning framework to reduce the labeling efforts for defect detection. The proposed framework adopts an iteration pattern to train the detection model. (2) To select effective data for annotations, we design an uncertainty sampling method to select images according their uncertainty values. (3) To confirm the sampling number for annotations, we design an average margin method to calculate the sampling ratios among defect categories. Extensive experimental results from a challenging public benchmark demonstrate the effectiveness of the proposed active learning method. In the future, we will explore more effective query strategies to reduce the labeling efforts; the adaptation of active learning is also under consideration.

## Figures and Tables

**Table 1:** Comparison of Recall on the NEU-DET dataset. The best results are shown in boldface.

Recall	Type				
	Inclusion (In)	Patches (Pa)	Pitted Surface (Ps)	Scratches (Sc)	Data (%)
Random	0.8333	0.9322	0.6774	0.9394	50.0%
EN	0.8636	0.9322	0.6774	0.9394	31.6%
MS	0.9242	0.9322	0.4839	1.000	33.3%
Full	0.8788	0.8983	0.6129	0.9091	100.0%
Ours	0.8485	0.9153	0.7742	0.9091	21.7%

**Table 2:** Comparison of Precision on the NEU-DET dataset. The best results are shown in boldface.

Precision	Defect				
	Inclusion (In)	Patches (Pa)	Pitted Surface (Ps)	Scratches (Sc)	Data (%)
Random	0.1291	0.1672	0.0323	0.2583	50.0%
EN	0.1839	0.2696	0.0669	0.2925	31.6%
MS	0.1017	0.2183	0.0498	0.2409	33.3%
Full	0.1213	0.2180	0.0617	0.1899	100%
Ours	**0.1965**	**0.3396**	**0.0774**	**0.3614**	21.7%

**Table 3:** Comparison of Average Precision (AP) on the NEU-DET dataset. The best results are shown in boldface.

AP	Defect					
	Inclusion (In)	Patches (Pa)	Pitted Surface (Ps)	Scratches (Sc)	mAP	Data (%)
Random	0.5498	0.7498	0.3082	0.5658	0.542	50.0%
EN	0.6359	0.8314	0.1959	**0.8546**	0.629	31.6%
MS	**0.6874**	0.7944	0.1763	0.9104	0.642	33.3%
Full	0.6183	0.7284	0.2103	0.7720	0.582	100%
Ours	0.6390	**0.8269**	**0.3277**	0.7874	**0.645**	**21.7**%

**Table 4:** The performance obtained via the full data.

Types	Inclusion (In)	Patches (Pa)	Pitted Surface (Ps)	Scratches (Sc)
Recall	0.8788	0.8983	0.6129	0.9091
Precision	0.1213	0.2108	0.0617	0.1899
AP	0.6183	0.7284	0.2103	0.7720
